# A multicenter, prospective, inpatient feasibility study to evaluate the use of an intra-colonoscopy cleansing device to optimize colon preparation in hospitalized patients: the REDUCE study

**DOI:** 10.1186/s12876-021-01817-2

**Published:** 2021-05-22

**Authors:** Helmut Neumann, Melissa Latorre, Tim Zimmerman, Gabriel Lang, Jason Samarasena, Seth Gross, Bhaumik Brahmbhatt, Haleh Pazwash, Vladimir Kushnir

**Affiliations:** 1 Department of Gastroenterology and Hepatology, University Medical Center Mainz, Mainz, Germany; 2 Division of Gastroenterology and Hepatology, New York University Langone Medical Center, New York City, NY USA; 3 Division of Gastroenterology, Washington University in St. Louis, 660 South Euclid Avenue, Campus Box 8124, St. Louis, MO 63110 USA; 4 Division of Gastroenterology and Hepatology, Department of Medicine, University of California, Irvine, Orange, CA USA; 5 Division of Gastroenterology and Hepatology, Mayo Clinic, Jacksonville, FL USA; 6 Division of Gastroenterology, Valley Hospital, Ridgewood, NJ USA

**Keywords:** Pure-Vu system, Bowel preparation, Quality improvement, Colonoscopy preparation, Colonoscopy

## Abstract

**Background:**

High quality bowel preparation prior to colonoscopy can be difficult to achieve in the inpatient setting. Hospitalized patients are at risk for extended hospital stays and low diagnostic yield due to inadequate bowel preparation. The Pure-Vu System is a novel device intended to fit over existing colonoscopes to improve intra-colonoscopy bowel preparation. The objective of the REDUCE study was to conduct the first inpatient study to evaluate optimization of bowel preparation quality following overnight preparation when using the Pure-Vu System during colonoscopy.

**Methods:**

This multicenter, prospective feasibility study enrolled hospitalized subjects undergoing colonoscopy. Subjects recorded the clarity of their last bowel movement using a 5-point scale prior to colonoscopy. After one night of preparation, all enrolled subjects underwent colonoscopy utilizing the Pure-Vu System. The primary endpoint was improvement of colon cleanliness from baseline to post-cleansing with the Pure-Vu System as assessed by the improvement in Boston Bowel Preparation Scale (BBPS). An exploratory analysis was conducted to assess whether the clarity of the last bowel movement could predict inadequate bowel preparation.

**Results:**

Ninety-four subjects were included. BBPS analyses showed significant improvements in bowel preparation quality across all evaluable colon segments after cleansing with Pure-Vu, including left colon (1.74 vs 2.89; p < 0.0001), transverse colon (1.74 vs 2.91; p < 0.0001), and the right colon (1.41 vs 2.88; p < 0.0001). Prior to Pure-Vu, adequate cleansing (BBPS scores of ≥ 2) were reported in 60%, 62%, and 47% for the left colon, transverse colon, and right colon segments, respectively. After intra-colonoscopy cleansing with the Pure-Vu System, adequate colon preparation was reported in 100%, 99%, and 97% of the left colon, transverse colon, and right colon segments, respectively. Subjects with lower bowel movement clarity scores were more likely to have inadequate bowel preparation prior to cleansing with Pure-Vu.

**Conclusions:**

In this feasibility study, the Pure-Vu System appears to be effective in significantly improving bowel preparation quality in hospitalized subjects undergoing colonoscopy. Clarity of last bowel movement may be useful indicator in predicting poor bowel preparation. Larger studies powered to evaluate clinical outcomes, hospital costs, and blinded BBPS assessments are required to evaluate the significance of these findings.

*Trial registration* Evaluation of the Bowel Cleansing in Hospitalized Patients Using Pure-Vu System (NCT03503162).

## Introduction

Colonoscopy is considered the gold standard for evaluating patients with suspected colonic disease. A key factor in ensuring high quality colonoscopy is good colon preparation [1, 2]. However, in hospitalized patients, bowel preparation is often challenging. This is due to patients’ inability to tolerate the preparation, slow bowel transit in the setting of immobilization and acute illnesses, and use of motility-altering medications [3, 4]. Thus, hospitalization is associated with a higher likelihood for poor bowel preparation and this can lead to incomplete colonoscopies, missed pathology, adverse events, prolonged hospital stays and increased costs [1–3].

A retrospective analysis of 8,819 hospitalized patients Garber et al. reported up to 51% of patients had inadequate bowel preparation for colonoscopy that lead to at least one additional day of hospitalization [5]. Another study of 524 inpatients reported that 55.4% of patients had a fair (35.1%) or inadequate (22.3%) bowel preparation, after going through the standard prep regime. These preparation levels were associated with 1.7 and 2.4 additional days in the hospital respectively as compared to patients with good or excellent prep [6]. In the current pandemic associated with COVID-19 and the associated hospital resource constraints, measures that can decrease length of stay related to in-hospital colonoscopies may alleviate some of the burden on the healthcare system.

**Fig. 1:**
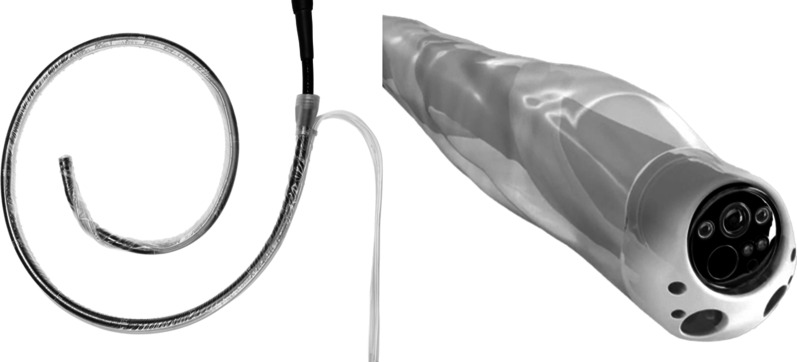
Pure-Vu® Sheath and Oversleeve

The Pure-Vu System (Motus GI® Holdings, Inc., Fort Lauderdale, FL) is a medical device with a 510(k) clearance by the U.S. Food and Drug Administration, and CE Mark approval that is used to help facilitate the cleansing of a poorly prepared colon during colonoscopy. The single use, disposable Oversleeve fits over the colonoscope (see Fig. 1) and is connected to a workstation. Two sizes of Oversleeve are available for use that are compatible with standard and pediatric/slim size colonoscopes. The System generates a mixture of water and air that creates a high intensity pulsed vortex that is delivered through four irrigation jets to break up fecal content. The fecal matter and fluids are simultaneously removed through two suction ports in the Oversleeve and delivered into external waste receptacles [7–9]. The Pure-Vu Workstation which is a control and pump system also has the ability to sense the buildup of material in the suction ports and automatically purge the ports to avoid clogging thus allowing significant debris to be removed from the colon.

A feasibility study including 47 outpatients at 3 clinical sites who were prescribed a minimal preparation of 20 mg Bisacodyl reported high rates of cecal intubation (97.9%), significant improvement in Boston Bowel Preparation Score (BBPS) scores (from a median score of 3.0 pre Pure-Vu to 9.0 post Pure-Vu cleansing), and high rates of physician satisfaction regarding system usability [8]. An additional study of 50 outpatients who were prescribed a minimal preparation of 20 mg Bisacodyl showed that Pure-Vu significantly increased BBPS scores and increased the proportion of patients with an adequate cleansing level (31% of patients at baseline to 98% post Pure-Vu cleansing; p < 0.001) [9].

Although there are data on the safety and efficacy of the Pure-Vu System in outpatients undergoing colonoscopy, there are no data on the use of this system to optimize bowel preparation in hospitalized patients undergoing colonoscopy. We hypothesized that the colon cleansing level post Pure-Vu procedure would be significantly improved as compared to the baseline colon preparation level (prior to Pure-Vu use) in hospitalized patients.

## Study design

This was a prospective, multicenter, cohort feasibility study to evaluate the performance and safety of the Pure-Vu System in hospitalized subjects. Subjects were enrolled at five clinical sites in the United States and one clinical site in Germany. The study protocol was approved by the local institutional review board (IRB) or ethics committee (EC) of all six participating medical centers. The study was performed in compliance with the Declaration of Helsinki and registered on ClinicalTrials.gov (NCT03503162; first posted on 19/04/2018).

### Study population

Hospitalized subjects ≥ 22 years of age and who were scheduled to undergo colonoscopy were recruited. Exclusion criteria were as follows: known or suspected inflammatory bowel disease (IBD), suspected / known active diverticulitis, suspected / known bowel obstruction, ascites, Child Pugh C cirrhosis, recent organ transplantation (within past 30 days), those actively receiving intravenous inotropic medications, subjects with left-ventricular assist device (LVAD), known coagulation disorder (INR ≥ 2 or platelets < 50,000), hemodynamic instability based on health care provider judgement, pregnancy or breast feeding, altered mental status/inability to provide informed consent, participation in another clinical study in the last 2 months.

### Study procedures

Subjects underwent bowel preparation per usual individual hospital routine. Bowel preparation was not dictated by the study protocol, however, pre-colonoscopy bowel preparation typically included diet restrictions and 4 L of GoLYTELY® [Braintree Laboratories, Inc., Braintree, MA, USA] within the 24 h prior to their colonoscopy.

**Fig. 2:**
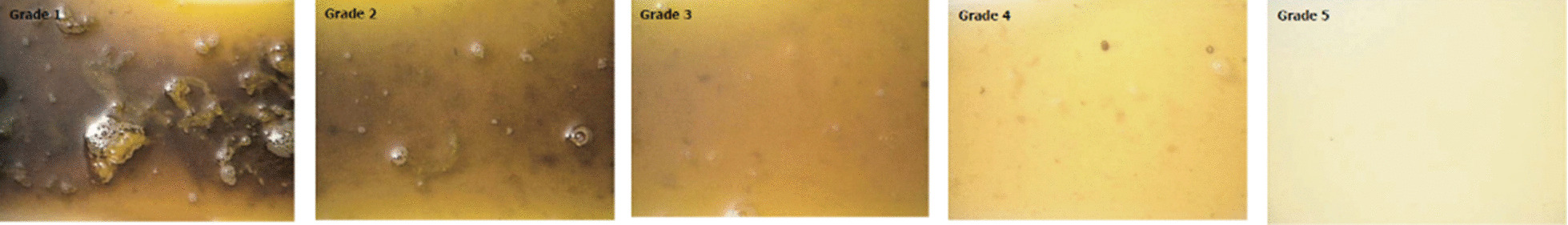
Reference Scale used to evaluate clarity of the last bowel movement prior to colonoscopy using the 5-point Kakugawa Clarity Scale (subject self-assessment)

Written informed consents from individual subjects were obtained and underwent colonoscopy the following day regardless of the amount of purgative ingested or clarity of the effluent from their last bowel movement. Baseline demographics including gender, age, body-mass-index, and indication for colonoscopy were obtained prior to colonoscopy. In addition, subject self-assessment of the clarity of the last bowel movement prior to colonoscopy was recorded using the 5-point Kakugawa Clarity Scale (see Fig. 2 for the clarity scale) [10]. All enrolled subjects underwent colonoscopy utilizing the Pure-Vu System to enhance bowel cleanliness regardless of the level of baseline bowel preparation. Each study subject was intubated once to obtain the endoscopist-determined baseline and post cleansing scores for all evaluable colonic segments. Upon reaching each segment, a baseline BBPS score was determined. After intra-colonoscopy cleansing with the Pure-Vu System, a second BBPS score was recorded for that segment. Therefore, the BBPS score was recorded for each colorectal segment (left colon, transverse colon, and right colon segments) both prior to (baseline) and after colon cleansing with Pure-Vu. Patients were monitored during and after the procedure per standard protocol by the investigator. Additionally, the patients were contacted by the study coordinator 48 h post-procedure to identify any delayed adverse events.

Prior to participating in the study all endoscopists had limited clinical experience using the Pure-Vu system with most endoscopists having performed only two to four procedures with the system. The decision to use an adult colonoscope versus pediatric/slim colonoscope with Pure-Vu System was at the discretion of the endoscopist.

### Study endpoints

The study’s primary endpoint was the rate of improved bowel cleansing level from baseline to after use of the Pure-Vu System per evaluable colon segment using the BBPS [11, 12]. An adequate cleansing level was a priori defined as a BBPS ≥ 2 in all evaluated colon segments.

Secondary endpoints included cecal intubation rate, the proportion of subjects with successful colonoscopy (defined as complete procedure conducted as scheduled for the intended indication), and safety.

### Statistical analyses

Subjects’ demographics and other baseline characteristics were reported as mean and SD for continuous variables or as median and interquartile range (IQR) for categorical variables. The McNemar test was used to compare proportions of adequate bowel cleansing (colon segments ≥ 2 BBPS) before and after cleaning with the Pure-Vu System.

A post-hoc, exploratory analysis was conducted to assess the relationship of self-reported stool clarity score in predicting the proportion of subjects with at least one inadequately prepared colonic segment (BBPS < 2) prior to using the Pure-Vu System. A likelihood ratio Chi-square test was used to assess statistical significance.

This was a feasibility study therefore no statistical considerations were made to determine a requisite sample size.

## Results

### Subject demographics

Colonoscopy with the Pure-Vu System was performed on 95 in hospital subjects. One subject was excluded from analysis due to the unexpected finding of active ulcerative colitis during colonoscopy which was a study exclusion. Therefore, a total of 94 subjects (60% male, mean age 62 ± 13 years, mean body mass index 28.0 ± 6.6) were included from 6 clinical study sites: Washington University Medical Center in St Louis, Missouri (n = 32), Mainz University Medical Center in Mainz, Germany (n = 25), NYU Langone Health in New York City, New York (n = 13), University of California Irvine, California (n = 12), the Mayo Clinic Medical Center in Jacksonville, Florida (n = 7) and Valley Hospital in Ridgewood, New Jersey (n = 6). Baseline subject demographics and indications for colonoscopy are summarized in Table 1.

**Table 1:** Baseline characteristics

	N = 94
Male, % (n)	60% (n = 56)
Age, mean (SD)	62 (13)
Body mass index, mean (SD)	28.02 (6.62)
Indications for colonoscopy, % (n)^a^	
Gastrointestinal bleeding	69% (n = 65)
Iron deficiency anemia	30% (n = 28)
Suspected neoplasia/colorectal cancer	12% (n = 11)
Abdomen pain/diarrhea/weight loss	10% (n = 9)
Other^b^	15% (n = 14)
Clarity of lase bowel movement prior to colonoscopy, % (n)	N = 93
Grade 1 (dirtiest)	2% (n = 2)
Grade 2	24% (n = 22)
Grade 3	25% (n = 23)
Grade 4	33% (n = 31)
Grade 5 (cleanest)	16% (n = 15)

^a^Patient may have more than indication for the colonoscopy procedure

^b^Evaluation for transplantation (n = 7), suspected lesion in colon/abnormal results on radiological imaging (n = 6), removed foreign object (n = 1)

### Pre-colonoscopy characteristics

Prior to colonoscopy, subjects reported the appearance of their last bowel movement using the 5-point Kakugawa Clarity Scale to describe stool clarity (Grade 1 (2%- dirtiest), Grade 2 (24%), Grade 3 (25%), Grade 4 (33%) and Grade 5 (16%- cleanest) Table 1).

### Colonoscopy characteristics

Colonoscopy was performed with an adult size colonoscope with Pure-Vu System Oversleeve in 53 subjects (56.4%) and with a pediatric/slim size colonoscope with Pure-Vu System Oversleeve in 41 subjects (43.6%). The total mean ± SD procedure duration was 27.4 ± 14.1 min.

### Primary endpoint: efficacy of Pure-Vu intra-colonoscopy cleansing

**Fig. 3:**
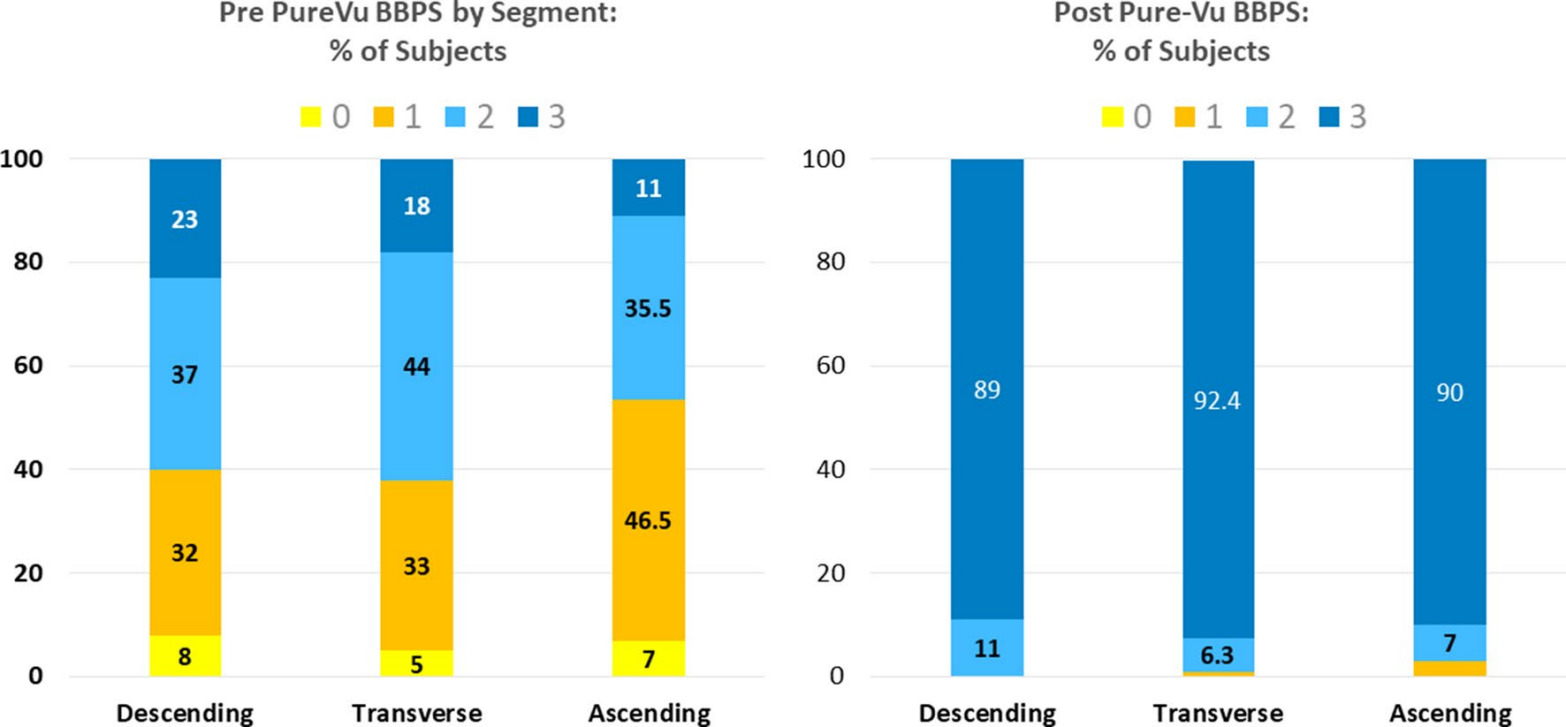
Pre and post Pure-Vu scores: % of subjects

The proportion of subjects with an adequate colon cleansing level (BBPS ≥ 2 in all of the evaluated colon segments), increased significantly from 38% prior to Pure-Vu cleansing to 96% following colon cleansing with the Pure-Vu System (p < 0.001). When evaluating individual colonic segments, the pre Pure-Vu BBPS scores of ≥ 2 were 60%, 62%, and 47% for the left colon, transverse colon, and right colon segments, respectively. After intra-colonoscopy cleansing with the Pure-Vu System, adequate colon preparation (BBPS ≥ 2) was reported in 100%, 99%, and 97% of the left colon, transverse colon, and right colon segments, respectively (Fig. 3). The Pure-Vu System significantly increased the BBPS mean scores across all three colonic segments (Table 2).

**Table 2:** Primary endpoint analysis of mean BBPS per evaluable colon segment

Mean BBPS	Pre Pure-Vu use	After Pure-Vu use	P value
Descending colon, Sigmoid and rectum, left (N = 84)	1.74 ± 0.91	2.89 ± 0.31	< 0.0001
Transverse colon (N = 78)	1.74 ± 0.81	2.91 ± 0.33	< 0.0001
Ascending and cecum, right (N = 73)	1.41 ± 0.71	2.88 ± 0.41	< 0.0001

### Secondary endpoints

Proportion of subjects with successful colonoscopy: this endpoint, which was defined as complete procedure conducted as scheduled for the intended indication, was achieved in 91/94 (97%%) subjects. Three subjects had incomplete colonoscopies: two subjects due to looping and one subject due to hard impacted stool.

#### Cecal intubation

**Fig. 4:**
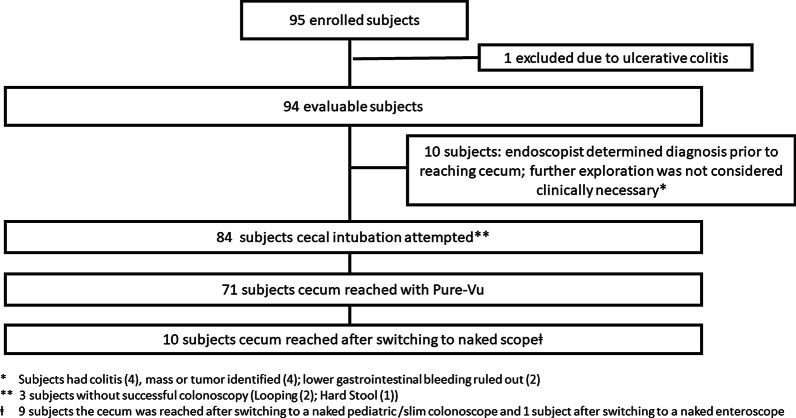
Patient flow/secondary endpoints

In 10/94 subjects (10.6%), the endoscopist determined the diagnosis prior to reaching the cecum due to encountering a mass/tumor, active colitis, ruling out the source of the bleed and further exploration of the colon was not considered clinically necessary. In 84 subjects cecum intubation was attempted. The cecum was reached in 71 subjects using the Pure-Vu Oversleeve which led to a cecal intubation rate of 75.5% (71/94) in the entire cohort and 84.5% (71/84) in subjects where cecal intubation was attempted. In an additional 10 (10.6%) subjects, the cecum was reached after switching to a naked pediatric/slim colonoscope or enteroscope. Therefore, the cecum was intubated in 81 total subjects which led to cecal intubation rate of 86.1% (81/94) in the entire cohort or 96.4% (81/84) in subjects where cecal intubation was attempted (See Fig. 4 for Patient Flow / Secondary Endpoints).

#### Safety

One serious adverse event was reported. One subject had a procedure-related, 1 cm rectal perforation, which occurred during attempted retroflexion. This required surgical repair. The subject was discharged 48 hours post operatively and fully recovered with no additional clinical sequelae. A total of three mild adverse events were also reported including fever, abdominal pain, and a reduction in hemoglobin from 7.6 g/dL pre-procedure to 6.6 g/dL. All three adverse events resolved, and the investigators recorded that the events were unlikely related to use of the Pure-Vu System.

#### Clarity of last bowel movement

**Fig. 5:**
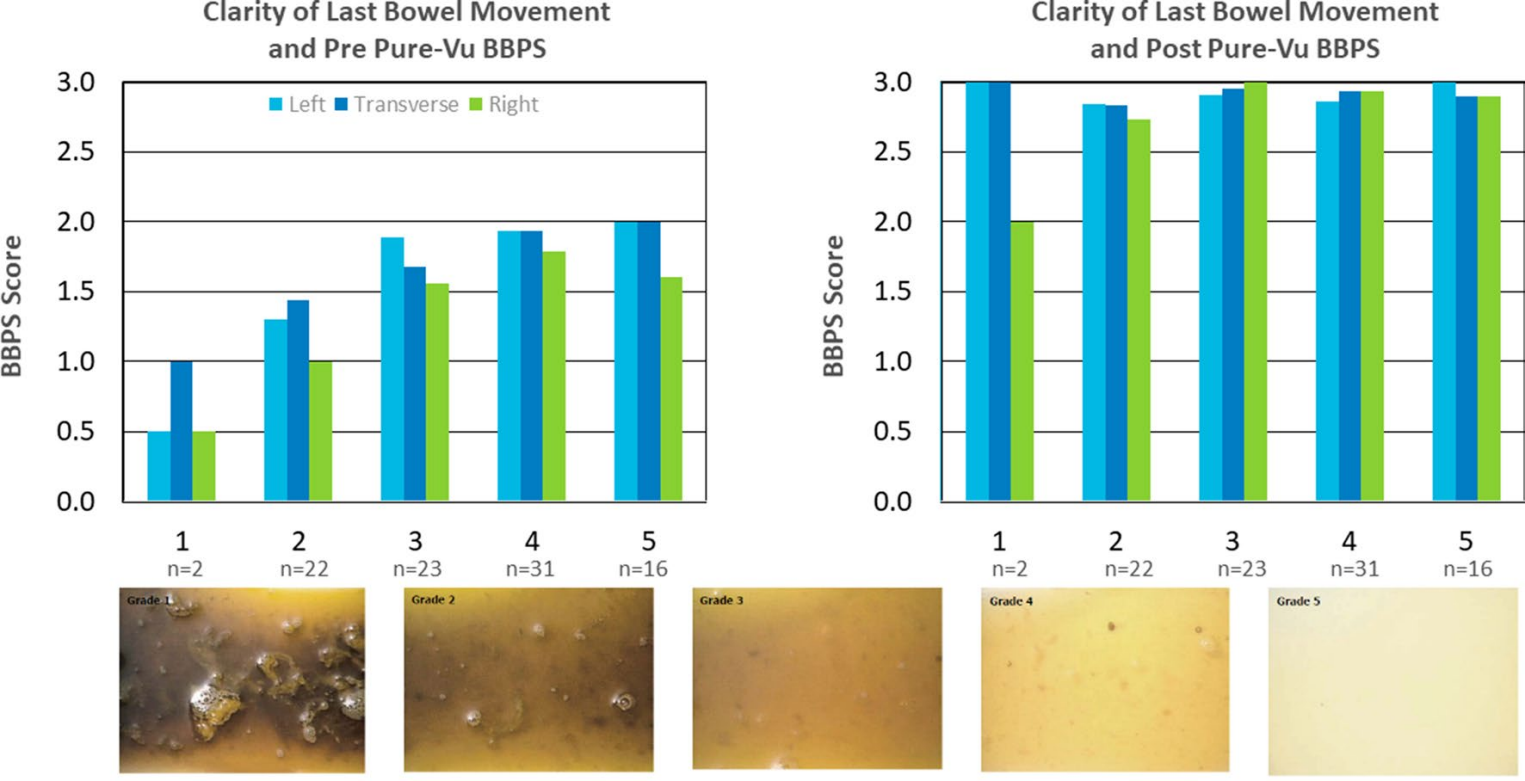
Pre and post Pure Vu scores by pre-procedure clarity of bowel movement

The subject’s self-report of last bowel movement clarity using the Kakugawa Clarity Scale was analyzed to evaluate possible association with the subject’s baseline bowel preparation prior to the use of Pure-Vu. We observed that 81% of subjects with a self-reported lower clarity score of 1 or 2 (poor clarity of last bowel movement) had at least one inadequately prepared colonic segment (BBPS < 2) (p = 0.03, OR 0.29, 95% CI 0.09–0.97). Additionally, 70% of subjects with a clarity score of 1, 2, or 3 had at least one inadequately prepared colonic segment, although this was not statistically significant (p = 0.09, 95% CI 0.19–1.14). The Pure-Vu System cleansing improved BBPS scores regardless of baseline clarity score (Fig. 5).

## Discussion

In this multi-center, international study which included 94 in-hospital subjects with diverse indications for colonoscopy, the Pure-Vu System significantly improved bowel cleanliness across all evaluable colonic segments and was able to facilitate a successful examination in 98% of patients in subjects sent for colonoscopy within 24 h of preparation regardless of volume preparation consumed or effluent clarity.

Prior to cleansing with the Pure-Vu System, 62% of patients had inadequate colon preparation defined as a BBPS < 2 in any single segment of the evaluated colon. This level of inadequate colon preparation is on the high end of what has been reported in the literature [5], likely due to the fact that per study protocol patients proceeded to colonoscopy as scheduled regardless of volume of bowel preparation consumed or consistency of fecal effluent.

In this study, 26% (24/94 subjects) of patients had a clarity score of two or less indicating dirty effluent prior to colonoscopy. The consistency of the effluent of the patients last bowel movement had a high correlation to at least one segment of the colon being inadequately prepped. This clarity score has the potential to be a predictive tool for identifying patients that may benefit from initiating the procedure with Pure Vu loaded on the scope to optimize the procedural success of the colonoscopy. Similar to a recently published prospective, single-blind, randomized trial, the volume of purgative consumed did not correlate to the level of preparation in the inpatient setting [13].

In the past decade, several systems have been introduced to the market to aid intraprocedural colon cleansing in patients who are found to be poorly prepared at the time of colonoscopy [14]. However, these devices have been limited by several factors: (1) they do not create multiple high intensity pulsatile water jets that can emulsify fecal matter, (2) They cannot simultaneously irrigate and suction debris from the colon, (3) they are used through the working channel of the colonoscope and can compromise the ability to suction fluid from the colon lumen, (4) the device has to be inserted and removed during the procedure in order to allow for evacuation of fluid and therapeutic maneuvers, thereby lengthening the procedure time. The Pure-Vu System overcomes these challenges by being an oversleeve placed over a standard colonoscope, thereby leaving the working channel free for instrument passage and suction. Moreover, the Pure-Vu System adds two additional evacuation channels, thus allowing for rapid removal of fecal matter and infused water during the colonoscopy procedure while creating a turbulent irrigation jet that causes significant agitation of the fluid within the colon to breakup fecal matter and dislodge fecal debris from the mucosa. Utilizing a high intensity intraprocedural bowel-cleansing device like the Pure-Vu System, which fits over a colonoscope, can enable endoscopists to improve visualization and facilitate the ability to reach a clinical diagnosis. Therefore, high intensity intraprocedural cleaning has the potential to improve quality of care, shorten hospital length of stay, reduce overall resource utilization on hospitals, and improve patient satisfaction.

One subject out of 94 subjects had a procedure-related, 1 cm rectal perforation, which occurred during attempted retroflexion with the Pure-Vu Oversleeve. While perforations are an uncommon occurrence and a serious event, inpatients undergoing colonoscopy are a higher risk population for perforation [15]. Finally, the instructions for use (IFU) for the Pure-Vu system includes a precaution to advance cautiously when performing rectal retroflexion.

Additional studies should be conducted with the Pure-Vu System in hospitalized patients to better understand the potential impact on clinical success, ability to reduce the need for repeat colonoscopy procedures, and to assess the impact on hospital resource utilization and length of stay. Devices such as the Pure-Vu System, which could improve quality of the exam and shorten the length of stay remain critically important to reduce the strain of hospital resources, which has become more critical than ever due to COVID-19. Moreover, self-assessment of pre-procedure bowel movement clarity may also be useful in identifying patients prior to colonoscopy who have a greater need for intra-procedural cleansing with the Pure-Vu System.

The limitations of this study include the lack of a control arm and a limited number of subjects to assess impact of improved bowel preparation on markers of clinical success (e.g., bleeding), hospital costs, and procedure time. While the study did not have a true control arm, the fact that quality of bowel preparation was evaluated during insertion of the colonoscope we feel that this serves as an internal comparator. However, assessments of pre- and post- Pure-Vu BBPS scores were not blinded and therefore may have been impacted by unconscious bias on the part of the investigators. In this study, colonoscopy was performed with a pediatric/slim colonoscope with Pure-Vu System Oversleeve in 43.6% of subjects. There may be some preference for endoscopists to use the more flexible pediatric/slim colonoscope with the Pure-Vu Oversleeve, but this information was not captured in the study.

Another limitation is that the administration of bowel preparation was not standardized between centers, given differences in formulary coverage and individual hospital protocols for colonoscopy preparation. The impact of this difference should be minimal however, since patients essentially served as their own controls as baseline bowel preparation was evaluated prior to cleansing, thus not impacting the primary endpoint.

In summary, the Pure-Vu System appears to be safe and effective in significantly improving bowel preparation quality in hospitalized subjects clinically indicated for colonoscopy. Pure-Vu could result in overall quality improvement for colonoscopy and optimize the time to a diagnosis in this population of hospitalized patients at high likelihood for inadequate bowel preparation.

## Data Availability

The datasets used and/or analysed during the current study are available from the corresponding author on reasonable request.

## References

[1] Harrison NM, Hjelkrem MC (2016). Bowel cleansing before colonoscopy: balancing efficacy, safety, cost and patient tolerance. World J Gastrointest Endosc.

[2] Baker FA, Mari A, Nafrin S (2019). Predictors and colonoscopy outcomes of inadequate bowel cleansing: a 10-year experience in 28,725 patients. Ann Gastroenterol.

[3] Ness RM, Manam R, Hoen H (2001). Predictors of inadequate bowel preparation for colonoscopy. Am J Gastroenterol.

[4] Jawa H, Mosli M, Alsamadani W (2017). Predictors of inadequate bowel preparation for inpatient colonoscopy. Turk J Gastroenterol.

[5] Garber A, Sarvepalli S, Burke CA (2019). Modifiable factors associated with quality of bowel preparation among hospitalized patients undergoing colonoscopy. J Hosp Med.

[6] Yadlapati R, Johnston ER, Gregory DL (2015). Predictors of inadequate inpatient colonoscopy preparation and its association with hospital length of stay and costs. Dig Dis Sci.

[7] Gross S, Gerson L, Lewis B (2018). A novel device for improving visualization in an inadequately prepared colon. Gastrointest Endosc.

[8] van Keulen KE, Neumann H, Schattenberg JM (2019). A novel device for intracolonoscopy cleansing of inadequately prepared colonoscopy patients: a feasibility study. Endoscopy.

[9] Pérez Jiménez J, Diego Bermúdez L, Gralnek IM (2019). An intraprocedural endoscopic cleansing device for achieving adequate colon preparation in poorly prepped patients. J Clin Gastroenterol.

[10] Kakugawa Y, Saito Y, Saito S (2012). New reduced volume preparation regimen in colon capsule endoscopy. World J Gastroenterol.

[11] Lai EJ, Calderwood AH, Doros G (2009). The Boston Bowel Preparation Scale: a valid and reliable instrument for colonoscopy-oriented research. Gastrointest Endosc.

[12] Calderwood AH, Jacobson BC (2010). Comprehensive validation of the Boston Bowel Preparation Scale. Gastrointest Endosc.

[13] Hernandez PV, Horsley-Silva JL, Snyder DL (2020). Effect of bowel preparation volume in inpatient colonoscopy. Results of a prospective, randomized, comparative pilot study. BMC Gastroenterol.

[14] Hernandez G, Gimeno-Garcia QE (2019). Strategies to improve inadequate bowel preparation for colonoscopy. Front Med (Lausanne).

[15] Hamdani U, Naeem R, Haider F (2013). Risk factors for colonoscopic perforation: a population-based study of 80118 cases. World J Gastroenterol.

